# The Role of Serial Fetal Echocardiography in Postnatal Surgical Decision-Making for Borderline Left Ventricle: A Case Report

**DOI:** 10.3390/pediatric18010018

**Published:** 2026-02-02

**Authors:** Andreea Cerghit-Paler, Dorottya Gabor-Miklosi, Iolanda Muntean, George-Andrei Crauciuc, Daniela Toma, Laura Beligan, Liliana Gozar

**Affiliations:** 1 Doctoral School, George Emil Palade University of Medicine, Pharmacy, Science and Technology of Târgu-Mureș, 540139 Târgu-Mureș, Romania; palerandreea@yahoo.com; 2 Department of Pediatrics III, George Emil Palade University of Medicine, Pharmacy, Science and Technology of Târgu-Mureș, 540139 Târgu-Mureș, Romania; iolanda.muntean@umfst.ro (I.M.); daniela.toma@umfst.ro (D.T.); liliana.gozar@umfst.ro (L.G.); 3 Emergency Institute of Cardiovascular Diseases and Transplantation, 540139 Târgu-Mureș, Romania; laurabeligan@yahoo.com; 4 Center for Advanced Medical and Pharmaceutical Research, George Emil Palade University of Medicine, Pharmacy, Science and Technology of Târgu-Mureș, 540139 Târgu-Mureș, Romania; andreicrauciuc@gmail.com

**Keywords:** borderline left ventricle, echocardiography, congenital heart disease, biventricular repair, ductal-dependent systemic circulation

## Abstract

**Background**: Borderline left ventricle represents a heterogeneous spectrum of congenital heart disease for which accurate prediction of suitability for biventricular versus univentricular circulation is often difficult. Serial fetal echocardiography may provide dynamic information to support postnatal decision-making. **Case Presentation**: We report the case of a fetus diagnosed at 32 weeks’ gestation with a borderline left ventricle, ventricular disproportion, hypoplastic left-sided structures, ductal-dependent systemic circulation, and a non-restrictive ostium secundum atrial septal defect. Serial fetal echocardiographic evaluations demonstrated stable left ventricular dimensions, preserved systolic function, impaired diastolic relaxation, and absence of endomyocardial fibroelastosis. Postnatal echocardiography confirmed hypoplastic aortic arch and coarctation. Following multidisciplinary evaluation, a biventricular repair strategy was selected. At 14 days of life, the patient underwent aortic arch reconstruction and partial atrial septal defect closure with preservation of a small therapeutic interatrial communication. Postoperative evolution was favorable, with progressive left ventricular growth and preserved function. At 2-year follow-up, echocardiography showed normalized mitral and aortic valve z-scores, good left ventricular systolic performance, and no evidence of myocardial fibrosis. **Conclusions**: This case highlights the value of serial fetal echocardiography in guiding individualized management of borderline left ventricle. Careful assessment of ventricular function and atrial septal physiology may support selection of a biventricular strategy in selected patients and contribute to favorable mid-term outcomes.

## 1. Introduction

Borderline left ventricle represents a heterogeneous spectrum of congenital heart disease characterized by varying degrees of hypoplasia of the left-sided cardiac structures, in which postnatal management remains particularly challenging [1]. Within this spectrum, the distinction between patients suitable for biventricular repair and those requiring univentricular palliation is often unclear, especially in the absence of critical mitral or aortic valve obstruction.

Despite significant advances in fetal cardiology, accurate prediction of postnatal circulatory suitability continues to be limited by the dynamic and multifactorial nature of left ventricular development. Several echocardiographic criteria and interventional strategies have been described for fetuses with critical aortic stenosis and duct-dependent systemic circulation, including fetal aortic valvuloplasty and early biventricular repair [2,3,4]. However, in cases of left ventricular hypoplasia without significant mitral or aortic stenosis, although multiple echocardiographic parameters and predictive models have been proposed to support postnatal surgical decision-making, no single marker or model has demonstrated consistent reliability across the heterogeneous spectrum of borderline left ventricular phenotypes [5].

A number of fetal echocardiographic markers have been proposed to support prognostication and parental counseling, including mitral valve z-scores, biphasic transmitral inflow patterns, flow characteristics across the foramen ovale, the presence of endomyocardial fibroelastosis, retrograde perfusion of the aortic arch, and anterograde transmitral flow [5,6]. More recently, increasing attention has been directed toward the role of the atrial septal defect (ASD) in the pathophysiology of borderline left ventricle, with accumulating evidence suggesting that ASD restriction may significantly influence left ventricular growth and function [7,8,9]. Accordingly, contemporary surgical strategies in selected patients increasingly favor a staged approach, allowing progressive conversion from univentricular palliation to biventricular circulation, with controlled restriction of a large ASD constituting an integral component of this management paradigm [10,11].

The aim of this report is to demonstrate how a physiology-driven, longitudinal assessment can support selection of a biventricular pathway in a fetus with severe left-sided hypoplasia traditionally considered unsuitable for biventricular repair. By prioritizing serial functional and hemodynamic trends over isolated anatomical thresholds, this case highlights the value of individualized decision-making and introduces a reproducible framework that complements conventional score-based models in borderline left ventricular disease.

## 2. Case Presentation

A pregnant woman was referred at 32 weeks’ gestation for specialized fetal cardiology assessment due to suspected congenital heart disease. The fetus showed appropriate intrauterine growth, with no extracardiac malformations detected on prenatal ultrasound. No invasive or non-invasive prenatal genetic testing had been performed during pregnancy.

### 2.1. Prenatal Echocardiographic Findings

**Figure 1:**
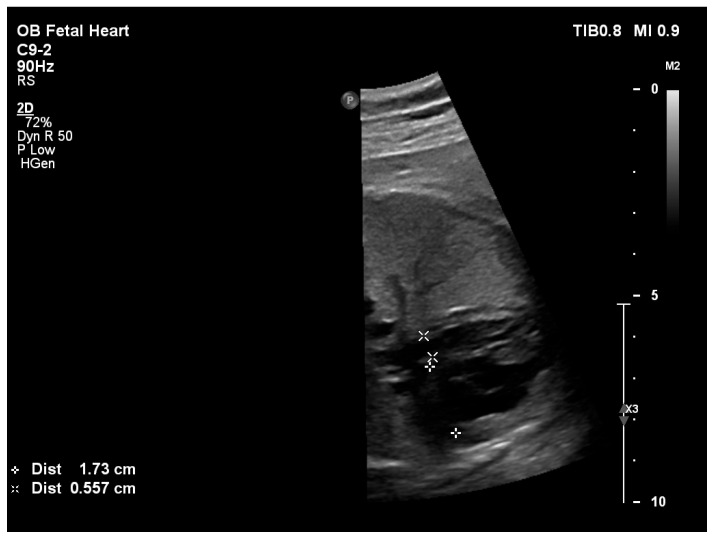
Four-chamber fetal echocardiographic view at 32 weeks’ gestation demonstrating measurement of the mitral valve annulus (0.557 cm) and tricuspid valve annulus (1.73 cm).

**Figure 2:**
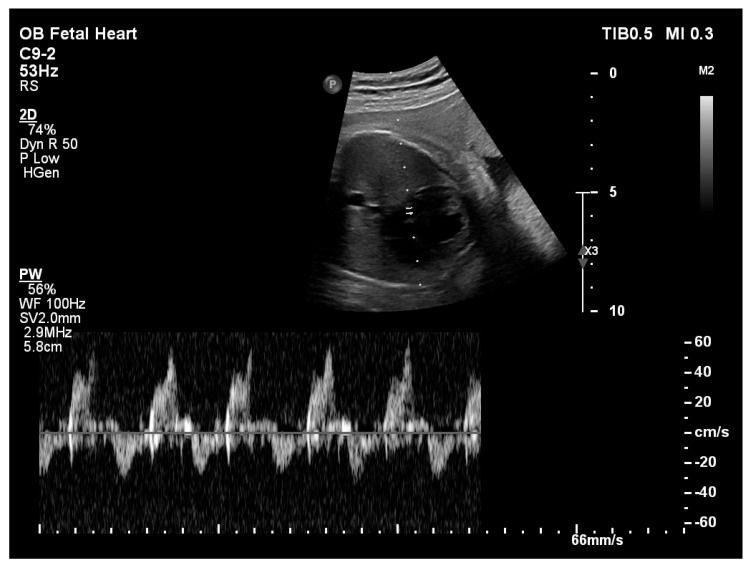
Spectral Doppler assessment of the mitral valve on fetal echocardiography showing a biphasic transmitral inflow pattern.

Fetal echocardiography at 32 weeks’ gestation demonstrated significant ventricular disproportion with marked dilation of the right-sided cardiac chambers and small left-sided cavities. A non-restrictive ostium secundum atrial septal defect was present. A non-restrictive atrial septal defect was defined as an interatrial communication of sufficient size to allow unrestricted atrial shunting, characterized by low-velocity Doppler flow and the absence of a significant pressure gradient across the atrial septum (mean gradient of 3 mmHg). The mitral valve annulus was hypoplastic, with a z-score [12] of −4.57 (Figure 1) and Doppler interrogation revealed a biphasic transmitral inflow pattern consistent with impaired left ventricular diastolic relaxation (Figure 2). The aortic valve annulus measured a z-score of −3.36.

**Figure 3:**
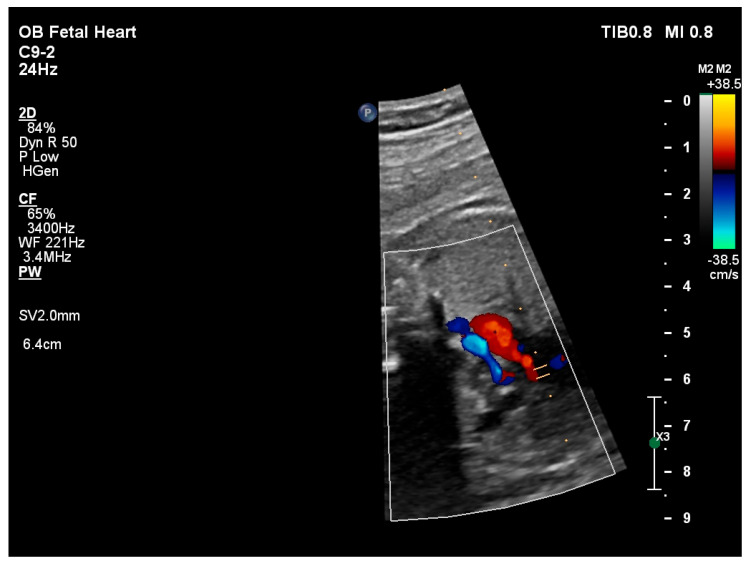
Three-vessel view on fetal echocardiography at 32 weeks’ gestation demonstrating retrograde filling of the ascending aorta.

Retrograde perfusion of the ascending aorta and aortic arch was observed (Figure 3), along with hypoplasia of the aortic isthmus (z-score −3.46), consistent with aortic coarctation. No endomyocardial fibroelastosis was identified.

Serial fetal echocardiographic evaluations were performed at 35 and 37 weeks’ gestation. Measurements of left-sided structures, transmitral flow pattern, and ventricular proportions remained relatively stable over time. In addition to standard fetal echocardiographic evaluation, left ventricular systolic function was serially assessed using fetal speckle-tracking echocardiography. Quantitative myocardial deformation analysis demonstrated preserved left ventricular systolic function throughout gestation, with stable values of left ventricular peak global longitudinal strain (LV pGLS) on serial examinations. These findings indicated maintained left ventricular contractile performance despite reduced chamber dimensions and the presence of diastolic dysfunction, providing complementary functional information beyond conventional morphological and Doppler assessment. Key echocardiographic parameters obtained during serial assessments are summarized in Table 1. Left ventricular volumetric assessment was performed using two-dimensional echocardiography at predefined fetal and postnatal time points. At 32 weeks’ gestation, fetal left ventricular end-diastolic volume (LVEDV) was 1.8 mL and left ventricular end-systolic volume (LVESV) was 0.8 mL, resulting in a calculated ejection fraction (EF) of 48.4%. At 34–36 weeks’ gestation, LVEDV measured 2.0 mL and LVESV was 1.2 mL, with a corresponding EF of 53.1%. Assessment for endomyocardial fibroelastosis was performed during serial fetal echocardiography using standardized qualitative morphological criteria. Particular attention was paid to the presence of increased endocardial echogenicity, diffuse endocardial thickening, abnormal myocardial–endocardial interface, and reduced endocardial excursion of the left ventricular walls. Across all fetal examinations the left ventricular endocardium appeared smooth and thin, without focal or diffuse hyperechogenic layers or features suggestive of endomyocardial fibroelastosis.

### 2.2. Delivery and Neonatal Course

The patient was delivered at 38 weeks’ gestation. The newborn was male, with a birth weight of 4130 g and a length of 57 cm. Postnatal adaptation was initially favorable, with preductal oxygen saturation of 96% and postductal saturation of 90%.

**Figure 4:**
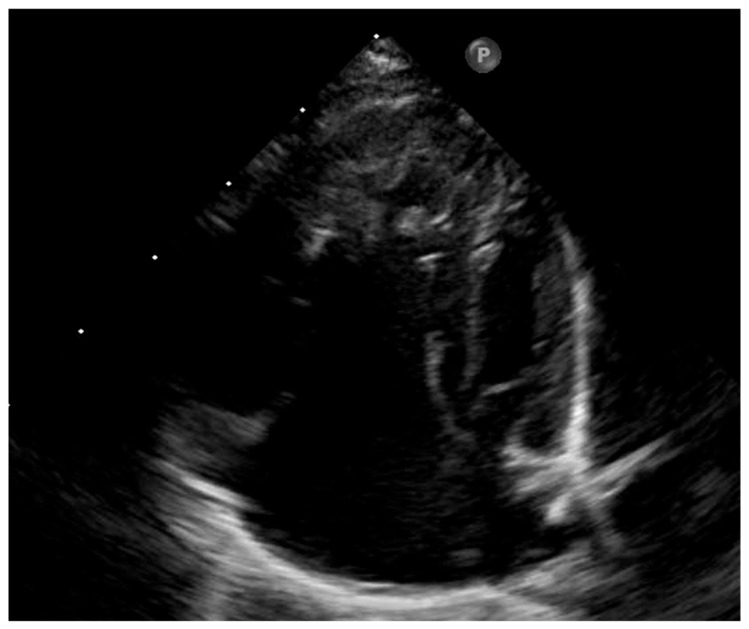
Neonatal echocardiographic four-chamber view showing marked dilation of the right-sided chambers, right ventricular dilation with hypertrophy, and hypoplastic left-sided cardiac cavities.

**Figure 5:**
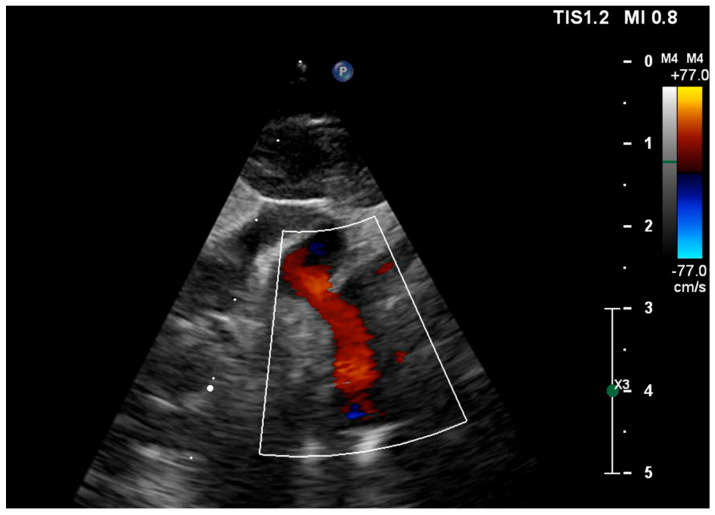
Neonatal echocardiography in the suprasternal view demonstrating retrograde perfusion of the aortic arch.

Postnatal transthoracic echocardiography confirmed ductal-dependent systemic circulation in the setting of a borderline left ventricle, hypoplastic ascending aorta and aortic arch, and aortic coarctation (Figure 4 and Figure 5), with retrograde filling of the aortic arch, however with antegrade flow in the ascending aorta. Calculated postnatal z-scores were −4.1 at the mitral valve annulus, −3.55 at the aortic annulus, −3.7 at the sinuses of Valsalva, −4.7 at the ascending aorta, −4.8 at the proximal aortic arch, −5.26 at the transverse arch, and −2.5 at the aortic isthmus. Transthoracic echocardiography demonstrated a left ventricular diastolic major axis of 23 mm and a systolic major axis of 18 mm. The postnatal left ventricular end-diastolic volume indexed to body surface area was 10 mL/m^2^. Additional findings included a bicuspid aortic valve, a patent ductus arteriosus with bidirectional shunting, and a non-restrictive ostium secundum atrial septal defect with left-to-right shunt. Left ventricular diastolic dysfunction with impaired relaxation was again noted. Continuous prostaglandin E1 infusion was initiated to maintain ductal patency.

### 2.3. Surgical Management

Following postnatal multidisciplinary evaluation, the decision to pursue a biventricular repair strategy was based on integrated echocardiographic findings rather than a single parameter. Key factors included the absence of endomyocardial fibroelastosis on serial fetal and postnatal imaging, preserved left ventricular systolic performance, stable left ventricular dimensions and volumes over time, non-restrictive atrial septal physiology, and the absence of critical aortic valve stenosis or atresia. At 14 days of life, the patient underwent heterologous pericardial patch augmentation of the ascending aorta, aortic arch, and proximal descending aorta, ligation of the patent ductus arteriosus, and partial closure of the atrial septal defect with intentional preservation of a small therapeutic interatrial communication. The procedure was performed under cardiopulmonary bypass.

### 2.4. Follow-Up

Postoperative hemodynamic evolution was favorable. Serial echocardiographic assessments demonstrated progressive growth of the left ventricle with preserved systolic function. Early postoperative echocardiography demonstrated left-to-right shunting across the residual interatrial communication (3 mm diameter) with a mean atrial-level gradient of 5 mmHg. No echocardiographic evidence of pulmonary venous congestion or progressive pulmonary hypertension was observed. Moderate mitral stenosis was present, with a mean transmitral gradient of 5 mmHg, and appropriate growth of the reconstructed aortic arch was observed.

**Figure 6:**
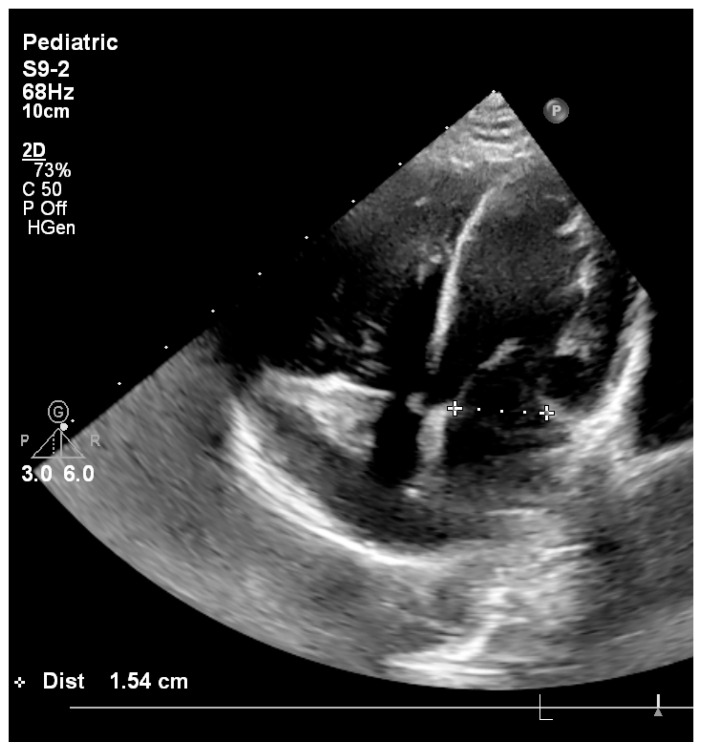
Follow-up transthoracic echocardiography demonstrating normalization of the left-sided cardiac chambers, with a mitral valve dimensions of 1.54 cm, 2 years after biventricular repair.

At 2-year follow-up, the patient exhibited normal somatic growth and remained asymptomatic, with no clinical signs of heart failure. Echocardiography showed normalized mitral annulus with a diameter of 15.4 mm and a z score of −0.6, with a mean transmitral gradient of 4 mmHg (Figure 6) and aortic valve of 10 mm witha a z-score of −1.3, preserved left ventricular systolic function quantified by speckle-tracking echocardiography (peak global longitudinal strain −21.9%), and absence of endomyocardial fibroelastosis. During the two-year follow-up period, diastolic performance was assessed using serial transthoracic echocardiography. Evaluation included transmitral Doppler inflow patterns, mean transmitral gradients, left atrial size, and indirect markers of pulmonary venous pressure and pulmonary hypertension. No progressive increase in transmitral gradients, left atrial dilation, or Doppler evidence of restrictive filling physiology was observed over time.

Serial postnatal echocardiographic assessment was documented in Table 2 and was used to evaluate left ventricular adaptation, diastolic tolerance, and atrial septal physiology following birth and surgical intervention. Parameters are presented longitudinally to emphasize postnatal hemodynamic evolution rather than isolated measurements.

### 2.5. Ethics

The present case report was approved by the Ethical Committee of the Emergency Institute of Cardiovascular Disease and Heart Transplantation from Targu Mures and “George Emil Palade” University of Medicine and Pharmacy Targu-Mures 3623/207.02.2025. Written informed consent was obtained from the patient’s legal guardians for publication of this case report and accompanying images.

## 3. Discussion

Fetal echocardiography plays a pivotal role in the prenatal diagnosis and longitudinal assessment of congenital heart disease, allowing for accurate anatomical characterization and functional evaluation throughout gestation [14,15]. In the specific context of borderline left ventricle, serial echocardiographic assessment is particularly important, as ventricular size, function, and loading conditions may evolve over time. Single time-point evaluations may therefore be insufficient to capture the dynamic nature of this condition and may lead to premature or inappropriate postnatal management decisions [5,6].

One of the central challenges in borderline left ventricle remains the selection between biventricular repair and univentricular palliation. Several echocardiographic predictors have been proposed to support this decision, including mitral and aortic valve z-scores, ventricular disproportion, transmitral inflow pattern, and the presence of endomyocardial fibroelastosis [5,6,15,16,17]. However, as highlighted in contemporary reviews, no single parameter has demonstrated adequate sensitivity or specificity when used in isolation [18]. In the present case, serial fetal assessments demonstrated relative stability of left-sided dimensions, preservation of left ventricular systolic function, and absence of endomyocardial fibroelastosis, all of which have been associated with favorable outcomes following biventricular repair. Serial fetal echocardiography was operationally defined as repeated standardized assessments performed at 32 and 34–36 weeks’ gestation, with comparison of left ventricular dimensions, volumetric indices, systolic function, and atrial septal physiology over time. Longitudinal analysis demonstrated relative stability of left ventricular size and volumes without evidence of progressive hypoplasia, growth deceleration, or in utero catch-up growth. This absence of interval deterioration, rather than absolute measurements at a single time point, was a key factor supporting the feasibility of a biventricular strategy. Left ventricular global longitudinal strain was not interpreted as a surrogate of ventricular volumetric adequacy or diastolic suitability for systemic circulation. pGLS primarily reflects intrinsic myocardial contractile performance and myocardial deformation, and does not directly assess ventricular compliance or filling capacity. In this case, pGLS was used as a complementary functional parameter, integrated with morphologic stability, atrioventricular valve dimensions, atrial septal physiology, and longitudinal evolution on serial fetal echocardiography. Importantly, the lack of progressive deterioration during late gestation supported the feasibility of a biventricular strategy.

Several validated predictive tools have been proposed to guide the selection of biventricular versus univentricular strategies in borderline left ventricle, including the Rhodes score, the Congenital Heart Surgeons’ Society (CHSS) regression equations, and the Boston criteria. The Rhodes score primarily integrates early postnatal echocardiographic measurements of left ventricular size, mitral and aortic valve dimensions, and ventricular performance to estimate suitability for biventricular repair, while the CHSS models focus on anatomical parameters and early survival outcomes following different surgical pathways. The Boston criteria emphasize left ventricular volume, mitral valve size, and the presence of endomyocardial fibroelastosis as key determinants of biventricular feasibility [2,3,4]. Applied retrospectively, the present case—characterized by a severely reduced mitral valve Z-score—would likely have been classified as unfavorable for primary biventricular repair by these threshold-based models. However, these scoring systems are largely derived from static, early postnatal datasets and do not incorporate longitudinal fetal functional data or evolving atrial septal physiology. In this case, the decision to pursue a biventricular strategy deliberately departed from score-based classification and was instead guided by serial fetal stability, preserved myocardial integrity, and a surgical plan designed to address anticipated diastolic constraints.

Recent literature has increasingly emphasized the role of atrial septal physiology in the evolution of borderline hypoplastic ventricles. Prenatally, restriction of the interatrial communication may lead to increased left atrial pressure and worsening left ventricular diastolic dysfunction, potentially impairing ventricular growth and compliance [7,8,9]. In selected severe cases, fetal intervention aimed at atrial septal decompression has been proposed to improve left ventricular filling and growth [19,20]. In the present case, the atrial septal defect remained non-restrictive throughout fetal life, possibly contributing to the preservation of left ventricular function observed during serial evaluations. Given the recognized risk of restrictive physiology after biventricular repair, surgical strategy intentionally included partial atrial septal defect closure with preservation of a small therapeutic interatrial communication. This approach aimed to balance left atrial decompression during the early postoperative period while promoting left ventricular preload and adaptation, thereby directly addressing the dominant diastolic mechanism of failure described in borderline left ventricle physiology.

We acknowledge that in patients with borderline left ventricular physiology, failure after biventricular repair is most frequently related to restrictive ventricular filling, elevated left atrial pressure, and secondary pulmonary hypertension rather than primary systolic dysfunction. Assessment of systolic deformation alone is therefore insufficient to predict postnatal hemodynamic tolerance of a biventricular circulation. In this context, preserved fetal pGLS was interpreted as a marker of maintained myocardial contractile reserve and absence of advanced myocardial disease (such as endomyocardial fibroelastosis), rather than as evidence of adequate ventricular volume or compliance. The stability of pGLS values on serial examinations further suggested the absence of progressive maladaptive remodeling during late gestation.

While atrial septal restriction may be detrimental during fetal life, postnatal hemodynamic conditions substantially modify the physiological role of the atrial septal defect. Left ventricular preload becomes highly dependent on left atrial pressure, which in turn reflects left ventricular diastolic compliance [21]. In patients with borderline left ventricle and impaired diastolic relaxation, a large non-restrictive atrial septal defect may become counterproductive by excessively decompressing the left atrium, reducing left ventricular preload and stroke volume, and activating compensatory neurohumoral mechanisms that further increase afterload and impair ventricular performance [21,22,23]. These considerations have led to growing interest in controlled restriction of the atrial septal defect as part of staged or primary biventricular repair strategies [10,11].

In this physiological context, maintaining a small, hemodynamically insignificant atrial septal defect following biventricular repair may represent a balanced approach. Such a “therapeutic” atrial communication can act as a safety valve during the early postoperative period, particularly in patients with limited left ventricular compliance, while avoiding excessive left atrial decompression. In selected cases, this strategy may also facilitate non-invasive estimation of left atrial pressure and provide insight into diastolic function during follow-up [21]. In the present patient, partial atrial septal defect closure with preservation of a small interatrial communication was associated with favorable early and mid-term hemodynamic adaptation.

Reintervention after biventricular repair in patients with borderline left ventricle remains common, with reported rates of 50–60% within the first postoperative year [24,25,26]. Early reintervention has been associated with increased morbidity and may reflect suboptimal initial patient selection. Echocardiographic markers associated with failure of biventricular repair include severely reduced mitral and aortic valve dimensions, left ventricular systolic dysfunction, and the presence of endomyocardial fibroelastosis [27,28]. In the present case, none of these high-risk features were identified, and mid-term follow-up demonstrated sustained left ventricular growth, preserved systolic function assessed by speckle-tracking echocardiography, and absence of myocardial fibrosis, supporting the appropriateness of the initial surgical decision.

Recent advances in fetal cardiology and congenital cardiac surgery have increasingly emphasized the potential role of fetal intervention and staged postnatal strategies in selected cases of borderline left ventricular physiology. Contemporary studies have demonstrated that fetal aortic valvuloplasty and other in utero interventions may promote left ventricular growth and improve the likelihood of achieving a biventricular circulation in carefully selected patients. In parallel, staged postnatal approaches aimed at controlled ventricular recruitment—often incorporating adaptive atrial septal management and delayed definitive repair—have been proposed as a means of balancing early hemodynamic stability with long-term biventricular potential. These evolving strategies underscore a shift away from rigid, threshold-based decision-making toward individualized, physiology-driven pathways that integrate serial imaging, myocardial integrity, and postnatal adaptive capacity. Awareness of these developments provides important context for interpreting borderline left ventricular phenotypes and reinforces the need for flexible, patient-specific management frameworks informed by both anatomy and longitudinal physiology [29,30,31,32].

Cardiac magnetic resonance imaging (CMR) is increasingly recognized as a valuable adjunct in the evaluation of patients with borderline left ventricular physiology, providing highly reproducible assessment of ventricular volumes, geometry, mass, and systolic function, and potentially improving risk stratification for biventricular versus univentricular repair. Several studies have shown that CMR-derived volumetric indices and ventricular geometry parameters may refine decision-making, particularly when echocardiographic findings are equivocal. However, neonatal CMR remains technically and logistically challenging, requiring dedicated neonatal scanners, specialized coils, advanced physiological monitoring, and anesthesia expertise. In the present case, CMR was not performed because our institution does not have dedicated neonatal CMR equipment. Anatomical and functional information was obtain from serial fetal and postnatal echocardiography. While CMR may further enhance individualized assessment in selected centers, echocardiography continues to represent the primary imaging modality for neonatal decision-making in many institutions [33,34,35].

Overall, this case underscores the importance of a multidisciplinary, physiology-driven approach in the management of borderline left ventricle. Rather than relying solely on static anatomical measurements, integration of serial echocardiographic data, functional assessment, and atrial septal physiology may allow for more individualized decision-making and improved outcomes. In line with recent recommendations, a staged and adaptable strategy appears particularly appropriate in cases where the optimal surgical pathway is not immediately evident [18].

Despite the integrative physiological framework presented, the relative weight of individual parameters (ventricular size, volumetric indices, atrial septal physiology, and functional markers) cannot be fully disentangled. As in many cases of borderline left ventricular disease, final commitment to a biventricular strategy relied on expert interpretation and institutional experience rather than on strictly reproducible thresholds. This limitation reflects the current state of evidence rather than a shortcoming specific to the present case.

### Limitations

The principal limitation of this report is its single-case design, which limits generalizability. Nevertheless, the detailed serial echocardiographic evaluation and favorable mid-term outcome provide clinically relevant insights into the role of dynamic assessment and tailored atrial septal management in patients with borderline left ventricle.

Advanced three-dimensional imaging modalities, such as CMR or computed tomography angiography, were not performed in this case. While these techniques may offer additional volumetric and anatomical detail, management decisions were based on serial fetal and postnatal echocardiography, reflecting current clinical practice in many centers.

Another limitation of this report is the relatively short duration of follow-up, which may not fully capture late hemodynamic changes, long-term ventricular remodeling, or valve-related outcomes following biventricular repair.

## 4. Conclusions

This case highlights the complexity of decision-making in fetuses with borderline left ventricular anatomy and underscores the limitations of relying on isolated anatomical thresholds or predictive scores. Serial fetal and postnatal echocardiographic assessment, focusing on longitudinal trends in ventricular size, myocardial integrity, atrial septal physiology, and functional performance, played a central role in guiding management. Although conventional scoring systems would have classified this patient as unfavorable for primary biventricular repair, an individualized, physiology-driven approach—combined with a risk-modulating surgical strategy—allowed for successful early biventricular adaptation. This report emphasizes that, in selected cases, careful integration of serial imaging, functional assessment, and staged surgical planning may support consideration of a biventricular pathway despite extreme anatomical constraints. Longer follow-up and larger studies are needed to better define which borderline phenotypes may benefit from such an approach.

## Figures and Tables

**Table 1:** Serial fetal echocardiographic parameters and literature-based reference thresholds.

Parameter	32 GW	35 GW	37 GW	Reference Ranges/Commonly Used Thresholds [2,3,4,12,13]
Ventricular disproportion (TV/MV ratio)	≥1.5 (Yes)	≥1.5 (Yes)	≥1.5 (Yes)	TV/MV ≥ 1.5 indicates significant ventricular disproportion
Mitral valve Z-score	−4.57	−4.40	−4.64	Normal: −2 to +2; Z-score < −3 associated with unfavorable anatomy for primary biventricular repair
Biphasic transmitral inflow	Present	Present	Present	Biphasic pattern consistent with impaired LV relaxation
Aortic annulus Z-score	−3.36	−3.30	−3.28	Normal: −2 to +2; Z-score < −3 indicates severe hypoplasia
Aortic isthmus Z-score	−3.46	−3.43	−3.40	Z-score < −3 consistent with severe arch hypoplasia/coarctation
Endomyocardial fibroelastosis	Absent	Absent	Absent	Presence of EFE is an adverse predictor for biventricular repair
Restrictive atrial septal defect	No	No	No	Restriction suggested by high-velocity atrial shunt and LA hypertension
Apex-forming left ventricle	No	No	No	Apex-forming LV generally favors biventricular feasibility
LV peak global longitudinal strain (%)	−18.3	−18.6	−19.3	Typical fetal LV pGLS ≈ −16% to −22%

GW: gestational weeks, MV: mitral valve, TV: tricuspide valve, LV: left ventricle, LV pGLS: left ventricular peak global longitudinal strain, EFE: endomyocardial fibroelastosis. Reference thresholds are derived from published fetal and neonatal echocardiographic studies [2,3,4,12,13] and are provided for contextual comparison rather than as absolute decision criteria.

**Table 2:** Serial postnatal echocardiographic parameters.

Parameter	First Day of Life	Day 1 Postoperative	2-Year Follow-Up
Mitral valve annulus (mm)	8.5	8.7	15.4
Mitral valve z-score	−4.1	−4.0	−0.6
Aortic valve annulus (mm)	5.9	6	10
Aortic valve z-score	−3.5	−3.5	+1.3
Left ventricle peak global longitudinal strain	−18.3%	−18.7%	−21.9%
Mean transmitral gradient (mmHg)	3	4	4
Atrial septal defect (mm)	6	3	3
Direction of atrial shunt	Left-to-right	Left-to-right	Left-to-right
Estimated pulmonary systolic pressure (mmHg)	54	42	20

## Data Availability

The raw data presented in this study can be obtained upon reasonable request addressed to Cerghit-Paler Andreea email address palerandreea@yahoo.com.

## Abbreviations

The following abbreviations are used in this manuscript:

LV	Left ventricle
LV pGLS	Left ventricular peak global longitudinal strain
ASD	Atrial septal defect
GW	Gestational weeks
TV	Tricuspid valve
MV	Mitral valve
EFE	Endomyocardial fibroelastosis.
CMR	Cardiac magnetic resonance
EF	Ejection fraction
LA	Left atrium
